# Validating biomarkers of chronic whiplash-associated disorders through magnetic resonance imaging techniques

**DOI:** 10.1186/s12998-025-00572-5

**Published:** 2025-05-04

**Authors:** Julia Evans, Michael Fishman

**Affiliations:** 1 Thomas Jefferson University, 1025 Walnut St #100, Philadelphia, PA 19107 United States; 2 Michael Fishman MD PLLC, 755 Norman Drive, Lebanon, PA 17042 United States

Dear Editor,

We read “Advanced magnetic resonance imaging of chronic whiplash patients: a clinical practice-based feasibility study” by Lars Uhrenholt et al. with great interest and commend their novel use of quantitative diffusion weighted imaging (DWI) and cerebrospinal fluid (CSF) flow magnetic resonance imaging (MRI) techniques to explore potential biomarkers for the chronicity of whiplash-associated disorders (WAD) [1]. We suggest a subsequent analysis of the cervical MRI data of this study to assess the proportion of cervical extensor muscles with fatty infiltrates. This approach has been shown to predict chronicity and severity of WAD and would be a straightforward follow-up analysis [2].

There is substantial evidence supporting the use of muscle fat infiltration (MFI) as a biomarker to distinguish chronic whiplash patients from healthy controls. Compared with healthy controls, a cohort of 79 individuals with chronic WAD had significantly greater fat infiltration in cervical extensor muscles, including the multifidus, semispinalis cervicis and capitis, splenius capitis, and upper trapezius [3].

However, the nuances of any correlation between cervical extensor muscle group atrophy and the unrecovered WAD phenotype remain uncertain. Compared with healthy controls, analyses of 31 and 30 individuals with chronic WAD found significantly greater MFI only in the right sternocleidomastoid and right trapezius, respectively, of the muscles assessed in each study [4, 5].

Given the small sample sizes in Uhrenholt’s study and the literature above, reevaluating the imaging of Uhrenholt’s cohort to compare MFI between chronic WAD patients and healthy controls could further establish or dispute the validity of this biomarker in evaluating cases of WAD, as well as further delineate the impact of chronic WAD on the fatty infiltration of particular muscles. Additionally, given the novelty of analyzing CSF flow dynamics and DWI in cases of chronic WAD, comparing this cohort to prior cohorts with significant correlations between MFI and chronic WAD may provide context for the validity of Uhrenholt’s null findings.

We believe there is substantial evidence that the cervical extensor MFI should have been a target calculated in the above cohort. We suggest that the authors of this innovative feasibility study reanalyze their MRI findings to calculate MFI. Similar to the protocol of Elliott et al. in 2006, MFI values of this cohort could be analyzed for group differences as well as for the potential influences of the Neck Disability Index (NDI), age, body mass index, and other collected data on fatty infiltration [3].

## Data Availability

No datasets were generated or analysed during the current study.

## Abbreviations

DWI: Diffusion weighted imaging
CSF: Cerebrospinal fluid
MRI: Magnetic resonance imaging
WAD: Whiplash-associated disorders
MFI: Muscle fat infiltration
NDI: Neck disability index
